# Design of uniform permanent magnet electronic optical system for 220 GHz sheet electron beam traveling wave tube

**DOI:** 10.1038/s41598-020-70016-y

**Published:** 2020-08-13

**Authors:** Wenxin Liu, Jiaqi Guo, Chao Zhao, Xin Guo, Meng Wang

**Affiliations:** 1 Key Laboratory of High Power Microwave Sources and Technologies, Aerospace Information Research Institute, Chinese Academy of Science, Beijing, 100190 China; 2 School of Electronic, Electrical and Communication Engineering, University of Chinese Academy of Science, Beijing, 100049 China

**Keywords:** Electronics, photonics and device physics, Plasma physics

## Abstract

The sheet electron beam (SEB), for which is the low current density and large current, is highly attractive in the region of millimeter wave and terahertz vacuum electronic devices (VEDs). A uniform permanent magnet (UPM) electronic optical system (EOS) driven by a SEB for 220 GHz traveling wave tube (TWT) is designed in present work, in which the voltage and current for SEB is 17 kV and 0.3 A, respectively. For obtaining the stable high transmission rate EOS, the characteristics of SEB in UPM EOS are studied, including the emittance, orbital angle, and beam trajectories, which are discussed through the CST simulation. The results show that the emittances in the *x*-direction are varied from 0.003 to 0.016 mm rad and in *y*-direction are various from 1 × 10^−4^ to 3 × 10^−4^ mm rad, respectively, keeping below than 2.5 × 10^−4^ mm rad during transmission, which guarantees the stability of SEB in *y*-direction. For the design of complete EOS, the normal rectangular collector is used, in which the SEB is uniformed scattering.

## Introduction

Recently, terahertz (THz, 1 THz = 10^12^ Hz) bands have been attracted many attentions for its myriad application^1^^,^ such as biochemical sensing, imaging for medical and security, astrophysics and remote atmospheric monitoring, and high-bandwidth communications, etc. Therefore, THz sources are highly focused on^2–4^, for which they are the key components of extensive applications, especial those with characteristics of wide band, high power and room temperature operation. Traveling wave tube (TWT) is one of the devices which can work at high frequency, and it is one of the most highlighted THz vacuum amplifiers due to its outstanding combined performances in bandwidth and power capacity^4^. In VED branch, the output power is scaled as the volume size, it will be sharply decreased with the increasing of operation frequency. On the other hand, the efficiency of beam-wave interaction is very low for the surface attenuation resulting by the light skin depth^5^.

As we know, the SEB is attracted and widely used to improve the output power in high frequency VED, such as the TWT, backward wave oscillator (BWO), and extended interaction klystron (EIK), etc. The SEB driving beam-wave interaction system of VED are widely appeared many literatures, Baig et al.^6^ analyzed the beam-wave interaction of 0.22 THz TWT driven by SEB with a help of CST simulation, and the high frequency characteristics are studied with cold measurements. Mark Field et al. developed a stagger-grating amplifier driven sheet beam, which produced the output power 107 W and a power-bandwidth product of 642 W GHz at centered frequency of 200 GHz^7^. The hybrid periodic permanent-magnet combined with the quadrupole magnet for a 220 GHz TWT is designed, the planar circuit is for interaction with a SEB with dimensions of 700 × 100 μm^2^ (7:1 aspect ratio)^8^. A SEB EOS for a G-band klystron is designed, the beam with a rectangular cross section of 2.5 mm × 0.15 mm is focused by uniform solenoidal magnetic field^9^. The difficulties of the SEB in VED EOS are the deflection, deformations and Diocotron instability during the transporting, it is resulted by the strong space charge force. To solve the technological difficulties, the strong confining magnetic field must be used, such as the UPM, period cusped magnet field, and wiggler, etc. For obtaining high power TWT, we use the UPM for the SEB EOS.

In present paper, we design a UPM EOS driven by SEB, in which the profile of UPM and the transmission characteristics of SEB are studied, including the emittance, orbital angle trajectories, section size and current are also analyzed. The rest paper is organized as follows: the design of SEB electron gun and electrostatic trajectories are described in second section, and the UPM field is designed in third section. The transmission characteristics of SEB in UPM are analyzed in fourth section, the rectangular collector for SEB is designed in fifth section and some conclusions are obtained in sixth section.

## Characteristics of electrostatic trajectory for SEB

**Figure 1:**
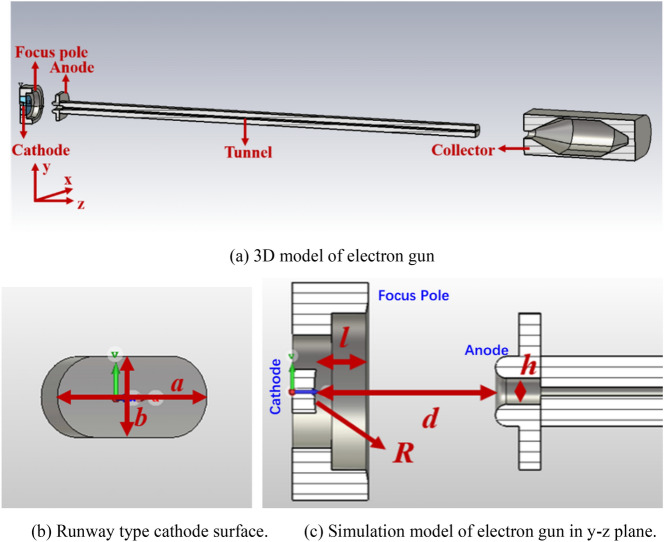
Simulation model of electron gun.

In present work, we use the Pierce electron gun structure for the design of UPM EOS^10^, there are consisted of cathode, focusing pole and collector, as shown in Fig. 1a. To generate a SEB, the runway shape of cathode surface is used as shown in Fig. 1b, in which the radius curvature *R* is selected as 3 mm, the cathode surface size *a* × *b* is 2 × 1 mm^2^. In the proposed 0.22 THz TWT, the voltage *U*, current *I* and the emission current density of SEB is 17 kV, 0.3 A, and 16.8 A/cm^2^, respectively, then the perveance is 0.135 μp. For the large emission current density, the scandic acid salt is used as the cathode emission material^11^. The focusing pole for the beam shaping, anode for accelerating of beam, magnetic field for the focusing of beam and collector for the recycling of interacted electron beam, the rectangular beam tunnel with height 0.2 mm is used to transport the SEB. The structure of focusing pole and anode is shown in Fig. 1c, here the length of focusing pole *l* is 1.7 mm, the distance between cathode and anode *d* is 4.4 mm, and the height of anode hole *h* is 0.6 mm.

**Figure 2:**
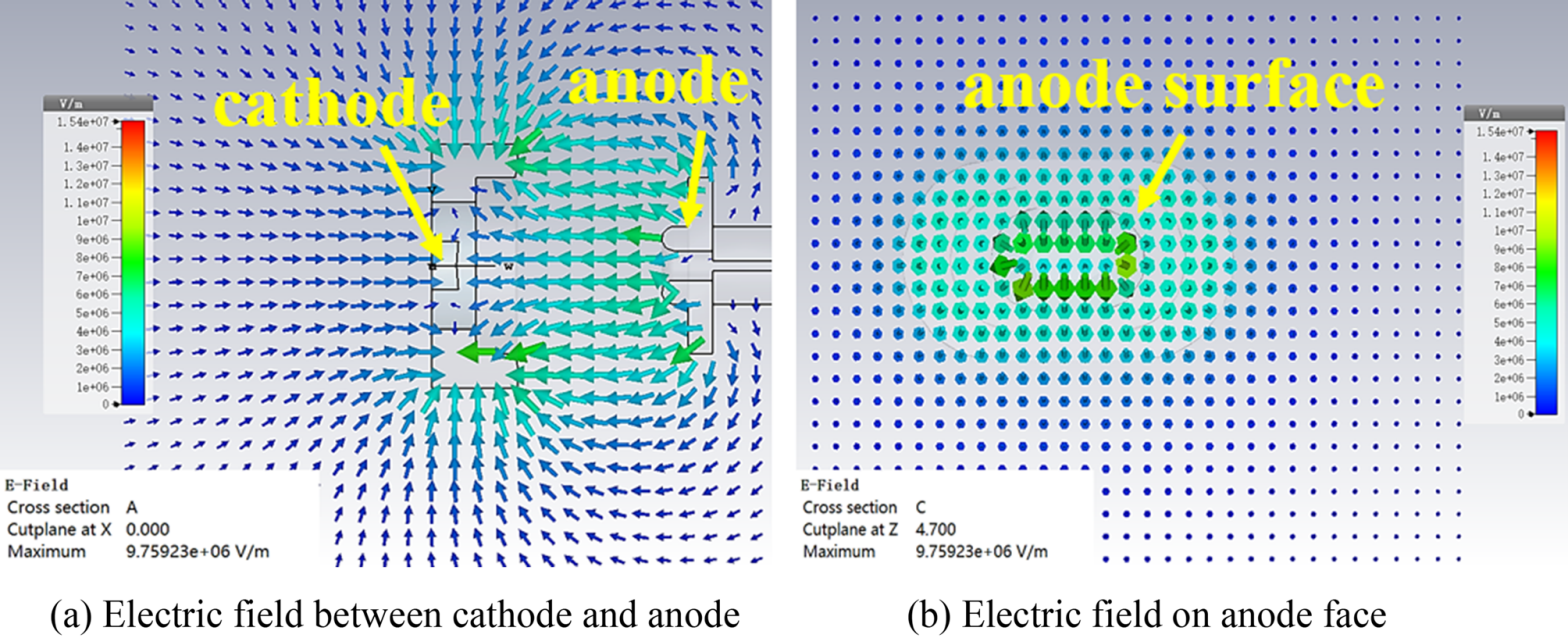
Electric field around gun.

**Figure 3:**
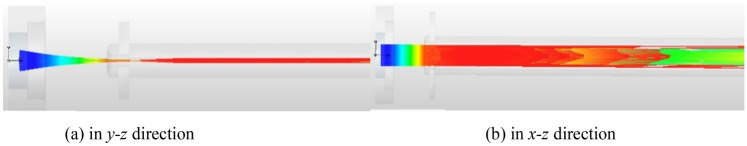
Electrostatic trajectory of electron beam (**a**) narrow side in *y–z* direction (**b**) wide side in *x–z* direction.

Based on the parameters above, the distributions of electric field around cathode and anode, in common with the electrostatic trajectory of SEB, are obtained by the CST studio shown in Figs. 2 and 3, respectively. Seen from the Fig. 2, the results show that the amplitude of electric field is the maximum when it locates between the cathode and anode. The cross side and longitudinal profile of SEB are shown in Fig. 3a,b, respectively. Obviously, the narrow side of SEB in the *y*-direction is compressed with the help of focusing pole, and the side is reduced to the thinnest at the beam waist, as shown in Fig. 3a. However, the wide profile of SEB in the *x*–*z* plane keeps the stable shape during the transporting as shown in Fig. 3b. The thickness of SEB at waist is compressed to be 0.035 mm, in which the total current is enclosed, and the width of SEB is kept at 2 mm.

**Figure 4:**
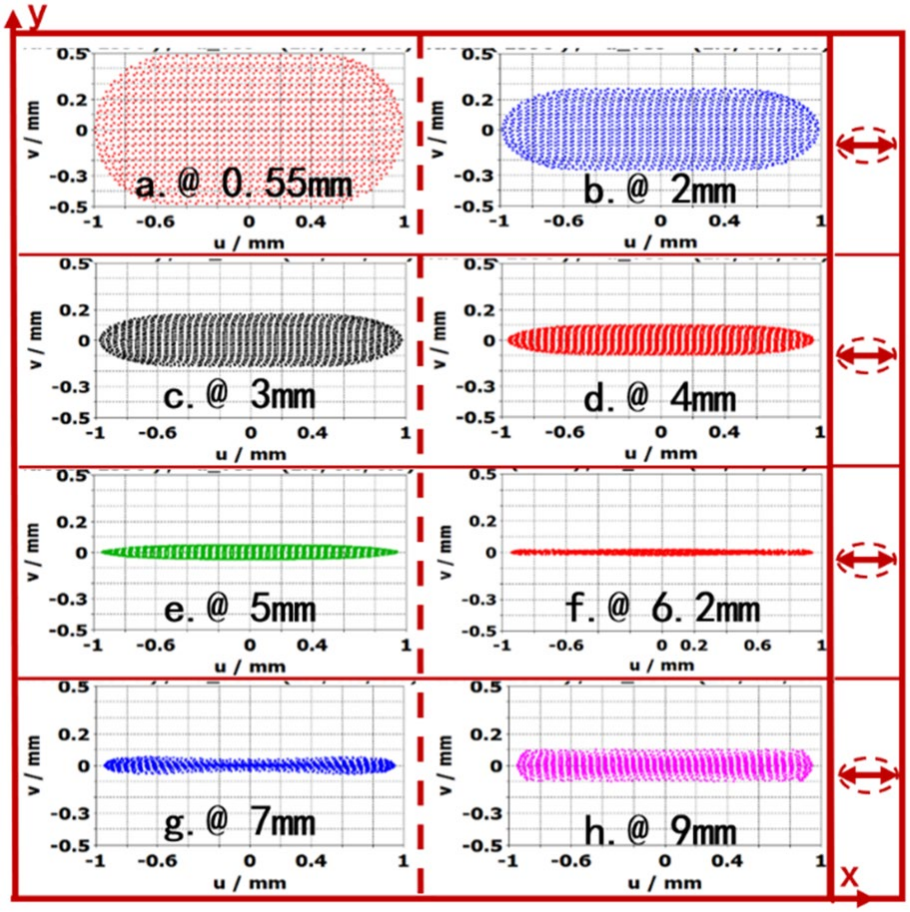
The cross size of electron beam at the longitudinal positions in *z*-direction.

To know the detailed changes of SEB size in the electrostatic EOS, the cross side of SEB at different longitudinal positions in *x*–*y* planes are shown in Fig. 4. The thickness of SEB in *x*–*y* plane is the thinnest at location of beam waist 6.2 mm, which is resulted from the combined interactions of focusing pole, cathode and anode. However, the width of SEB is kept with 2 mm from cathode to beam-wave interaction region.

**Figure 5:**
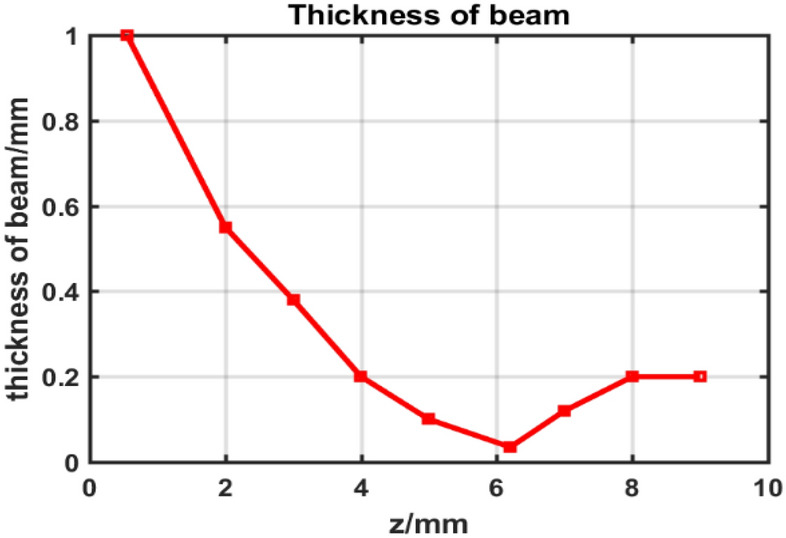
The thickness of sheet beam in z-direction.

Figure 5 shows the thickness of beam at the longitudinal position in *z*-direction from Fig. 4. The waist is located at 6.2 mm, the thickness of waist is 0.035 mm. The thickness of SEB is increased when transmission length is larger than that of waist, which is resulted from the space charge force. Therefore, to obtain a thin sheet beam, it is necessary to design a focus magnetic field to confine the electron beam transmission.

## Design of uniform permanent magnetic field

In VED field, there are three methods to confine and guide the electron beam transmission, one is the solenoid magnetic field, which is driven by the alternating current, and the profile of magnetic field is depended on the amplitude of current and turns. The second one is period permanent magnetic (PPM) field, this kind of PPM is suitable for the low current TWT, such as aerospace helix and coupled cavity TWT. And the third one is UPM, which is suitable for the large current and strong peak magnetic field. In present paper, we focus on the UPM consisted of neodymium iron boron (*NdFeB*) to confine the SEB EOS for TWT.

According to the electron beam parameters, the Brillouin magnetic field considered space charge effects can be calculated as^10^:

1 $${B}_{b}=\sqrt{\sqrt{2}{I}_{0}/4{r}_{x}{r}_{y}{\varepsilon }_{0}{\eta }^{3/2}{V}_{0}^{1/2}}$$

In which ${r}_{x}$ and ${r}_{y}$ are the half width and half thickness of SEB, respectively, then we calculated ${B}_{b}$ is 0.16* T*.

**Figure 6:**
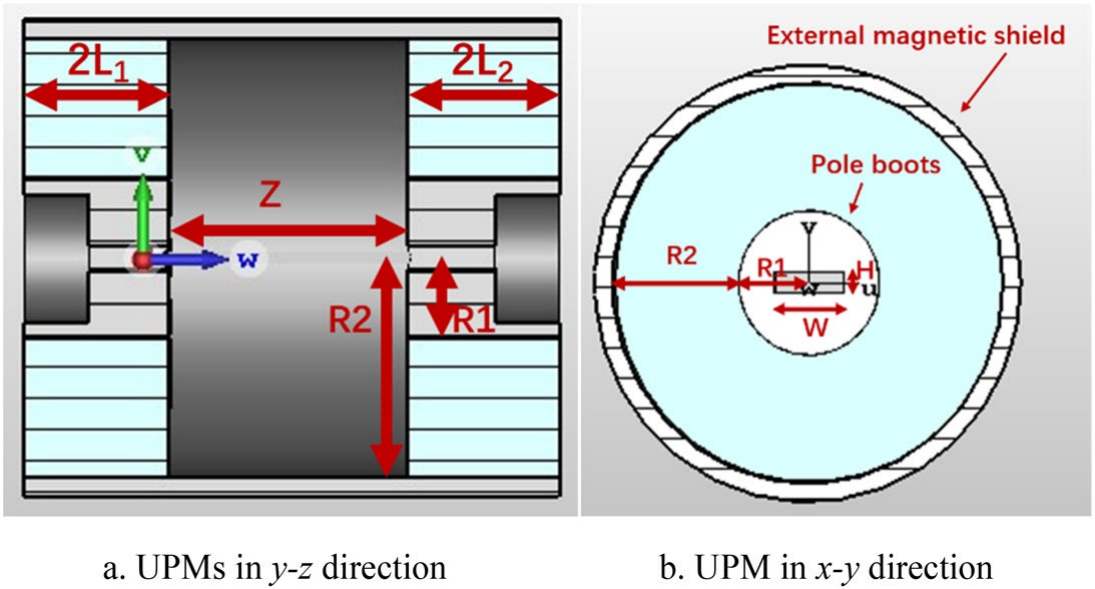
Simulation model of UPM.

The UPM structure consists of pole boots, magnets and external magnetic shield, the structure is shown in Fig. 6, the parameters of UPM are listed as Table 1.

**Table 1:** Parameters for UPM.

Items	Parameters (mm)
Thickness of first magnet (2*L*_1_)	36
Thickness of second magnet (2*L*_2_)	38
Distance between two magnets (*Z*)	60
Inner radius of magnet(*R*_1_)	20
Outer radius of magnet(*R*_2_)	55
Width of pole boots opening (*W*)	20
Height of pole boots opening (*H*)	6

The magnetic induction intensity at a point in the structure can be expressed as^12^:

2 $$\mathrm{B}\left(\mathrm{z}\right)=\frac{{B}_{r}}{2}\left[\frac{1}{{\mathrm{a}}_{1}}-\frac{1}{{\mathrm{a}}_{2}}-\frac{1}{{\mathrm{b}}_{1}}+\frac{1}{{\mathrm{b}}_{2}}+ln\frac{\left(1+{\mathrm{b}}_{1}\right)\left(1+{\mathrm{a}}_{1}\right)}{\left(1+{\mathrm{b}}_{2}\right)\left(1+{\mathrm{a}}_{1}\right)}\right]+\frac{{B}_{r}}{2}\left[\frac{1}{{\mathrm{a}}_{1}^{^{\prime}}}-\frac{1}{{\mathrm{a}}_{2}^{^{\prime}}}-\frac{1}{{\mathrm{b}}_{1}^{^{\prime}}}+\frac{1}{{\mathrm{b}}_{2}^{^{\prime}}}+ln\frac{\left(1+{\mathrm{b}}_{1}^{^{\prime}}\right)\left(1+{\mathrm{a}}_{2}^{^{\prime}}\right)}{\left(1+{\mathrm{b}}_{2}^{^{\prime}}\right)\left(1+{\mathrm{a}}_{1}^{^{\prime}}\right)}\right]$$

In which, ${a}_{1}=\sqrt{1+{\left(\frac{z+\frac{\mathrm{Z}}{2}+2{L}_{1}}{{R}_{2}}\right)}^{2}}$, ${a}_{2}=\sqrt{1+{\left(\frac{z+\frac{Z}{2}}{{R}_{2}}\right)}^{2}}$, ${b}_{1}=\sqrt{1+{\left(\frac{z+\frac{\mathrm{Z}}{2}+2{L}_{1}}{{R}_{1}}\right)}^{2}}$, ${b}_{2}=\sqrt{1+{\left(\frac{z+\frac{\mathrm{Z}}{2}}{{R}_{1}}\right)}^{2}}$, ${a}_{1}^{^{\prime}}=\sqrt{1+{\left(\frac{z-\frac{Z}{2}}{{R}_{2}}\right)}^{2}}$, ${a}_{2}^{^{\prime}}=\sqrt{1+{\left(\frac{z-\frac{\mathrm{Z}}{2}-2{L}_{2}}{{R}_{2}}\right)}^{2}}$, ${b}_{1}^{^{\prime}}=\sqrt{1+{\left(\frac{z-\frac{\mathrm{Z}}{2}}{{R}_{1}}\right)}^{2}}$, ${b}_{2}^{^{\prime}}=\sqrt{1+{\left(\frac{z-\frac{\mathrm{Z}}{2}-2{L}_{2}}{{R}_{1}}\right)}^{2}}$, 2*L*_1_ is the thickness of first magnet, 2*L*_2_ is the thickness of second magnet, and *Z* is the distance between two magnets, *R*_1_ is the inner radius of magnet, *R*_2_ is the outer radius of magnet.

**Figure 7:**
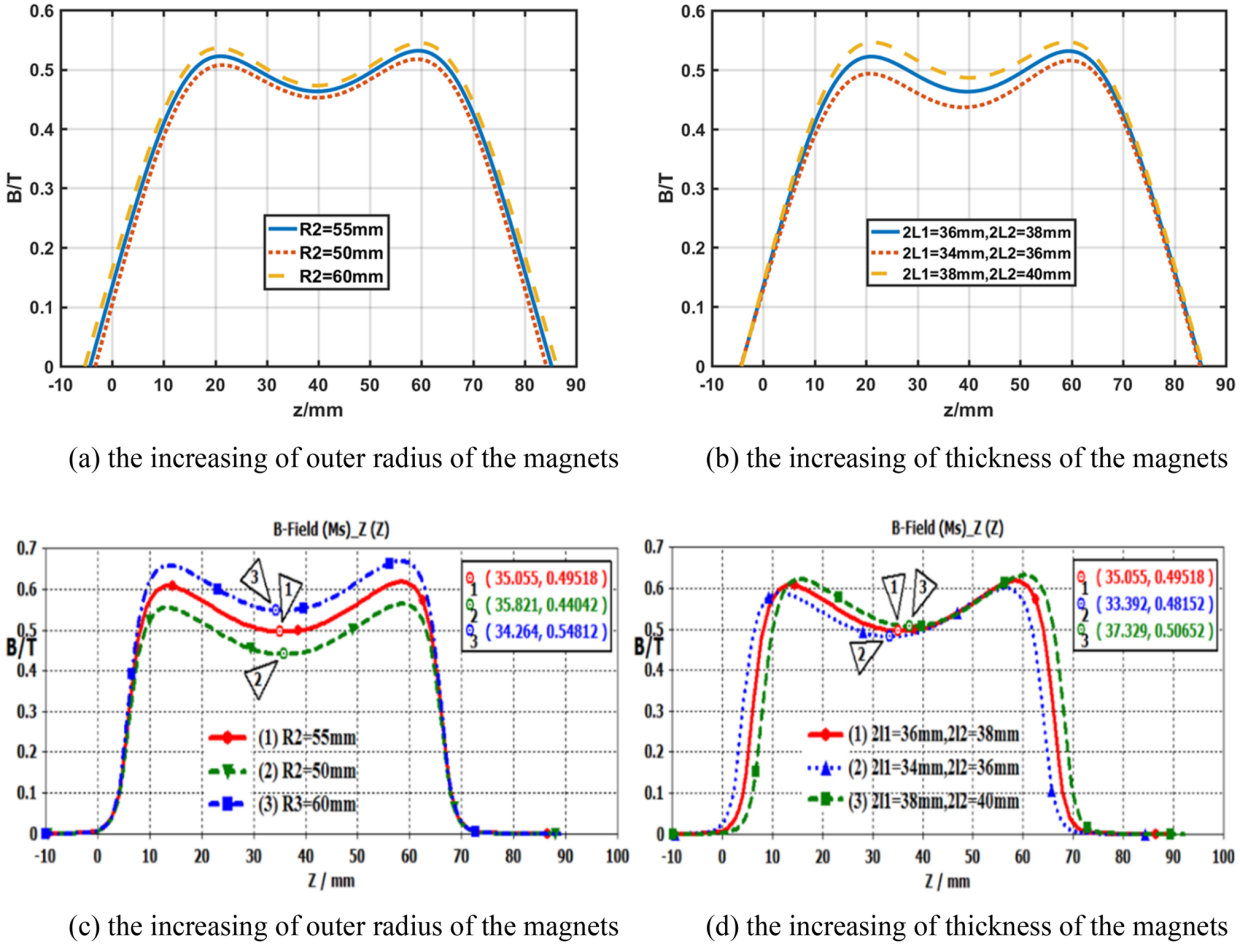
The profile of Longitudinal magnetic field intensity in z-direction produced by UPM.

Based on the formula (2), the distribution of magnetic induction field *B* in *z*-direction is shown in Fig. 7. The peak strength of *B* is 0.53 T in Fig. 7a and it is improved as the increasing of outer radius of the magnets, and Fig. 7b shows that the amplitudes of *B* are enhanced as the increasing of thickness of magnets. To verify the variations, the amplitude of *B* generated from theoretical calculations are compared with that of simulations, which are shown in Fig. 7c,d, and the results show that the theoretical calculations are consistent with those simulations.

**Figure 8:**
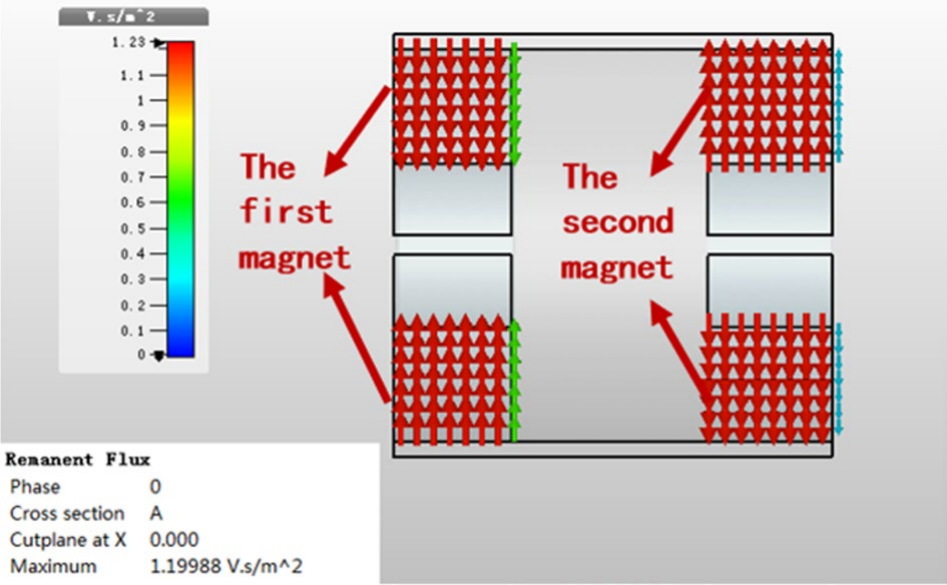
Remanent flux in y–z plane.

Figure 8 shows the distribution of magnetic remanent flux in *y*–*z* plane, from which we can see the maximum remanent flux intensity is 1.2 V s/m^2^, it is less than 2 V s/m^2^. The result shows that the profile of UPM aren’t reached the saturations.

**Figure 9:**
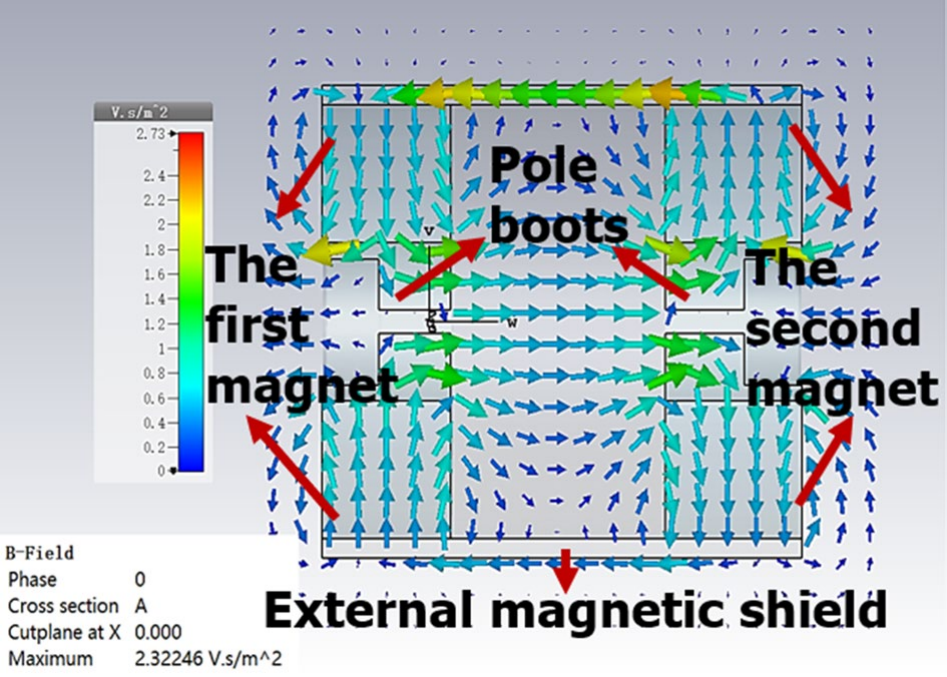
Distribution of magnetic vector in y–z plane.

Figure 9 shows the magnetic vector distribution of UPM. As we can see, the vectors of magnetic field in *z*-direction are parallel with the beam moving in longitudinal direction, and in the radial direction, the amplitude of B is very weak, it will benefit to the beam transmission.

## Transmission characteristics of SEB in UPM

### Electromagnetic trajectory under UPM

**Figure 10:**
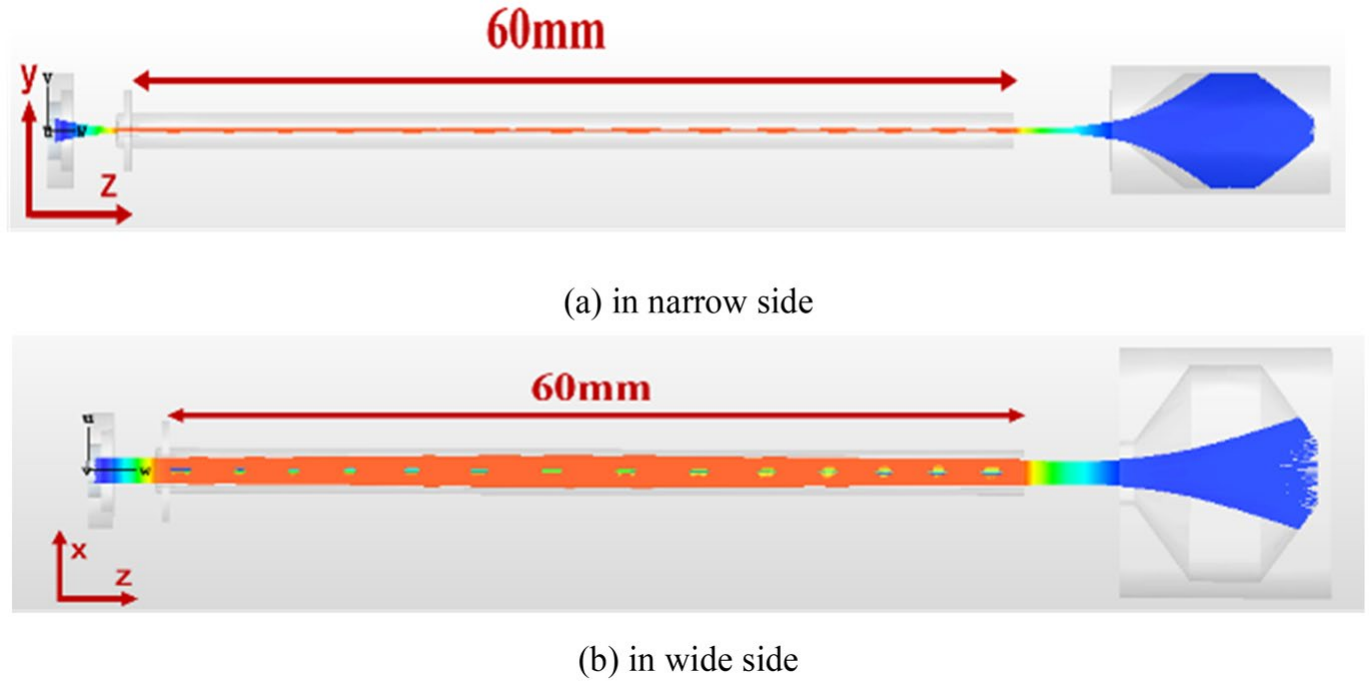
Trajectories of electron beam (**a**) in narrow side and (**b**) in wide side.

Based on the electrostatic trajectories, the electromagnetic one is obtained under the confining of UPM as shown in Fig. 10. Obviously, the SEB can stably transport length with 60 mm, however, the fluctuations are appeared in the *y*–*z* direction, the maximum and minimum of drift amplitude are 0.12 mm and 0.03 mm, respectively. The width of the electron beam increases first and then decreases in the wide side direction, with 2 mm at the beginning of the channel and 2.4 mm at the widest part.

**Figure 11:**
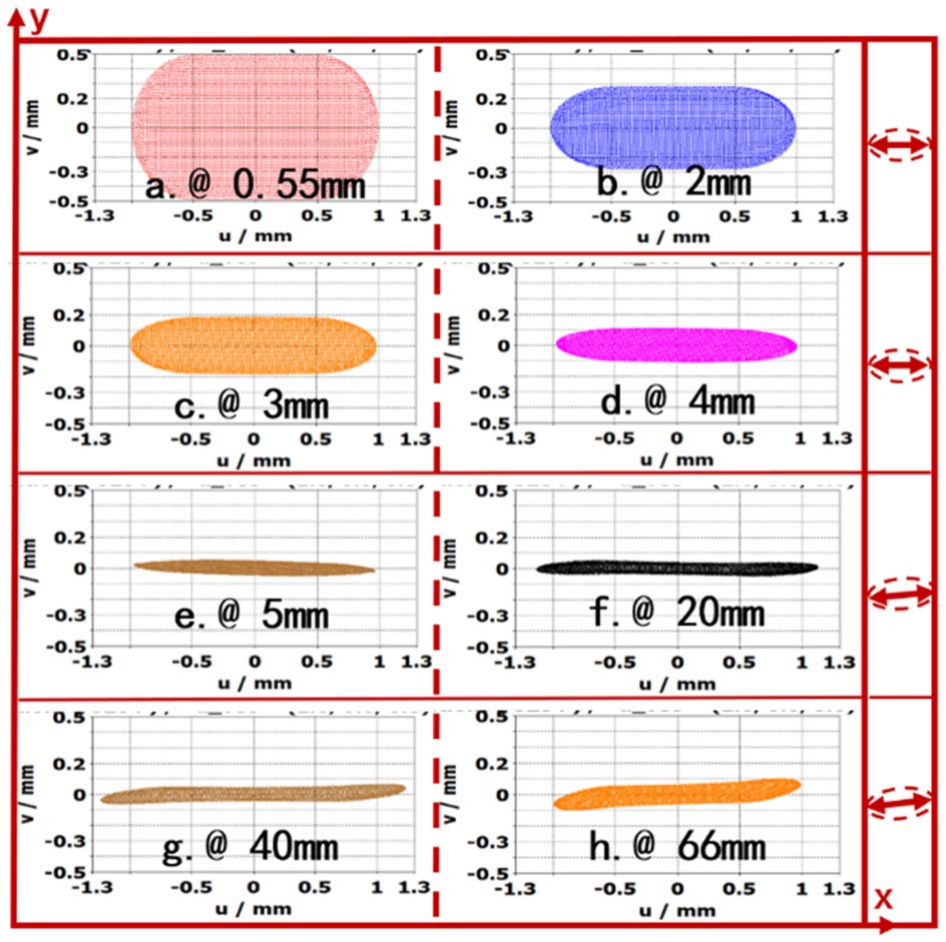
Electron trajectories cloud map at different positions in z-direction.

Figure 11 show the variations of cross size of SEB at different longitudinal positions. From the Fig. 11, the SEB is compressed after being emitted from cathode, the cross size is 0.08 mm × 2 mm at plane of 5 mm, and then transport for 60 mm keep at a section size of 0.08 mm × 2.4 mm, in which the total current is enclosed. The width of SEB changes to 2.4 mm and returns to 2 mm at the exit of tunnel. On the other hand, we find that the SEB is deflected in *y*-direction, but the deflection of trajectory is small under the UPM, which doesn’t affect the beam-wave interaction.

**Figure 12:**
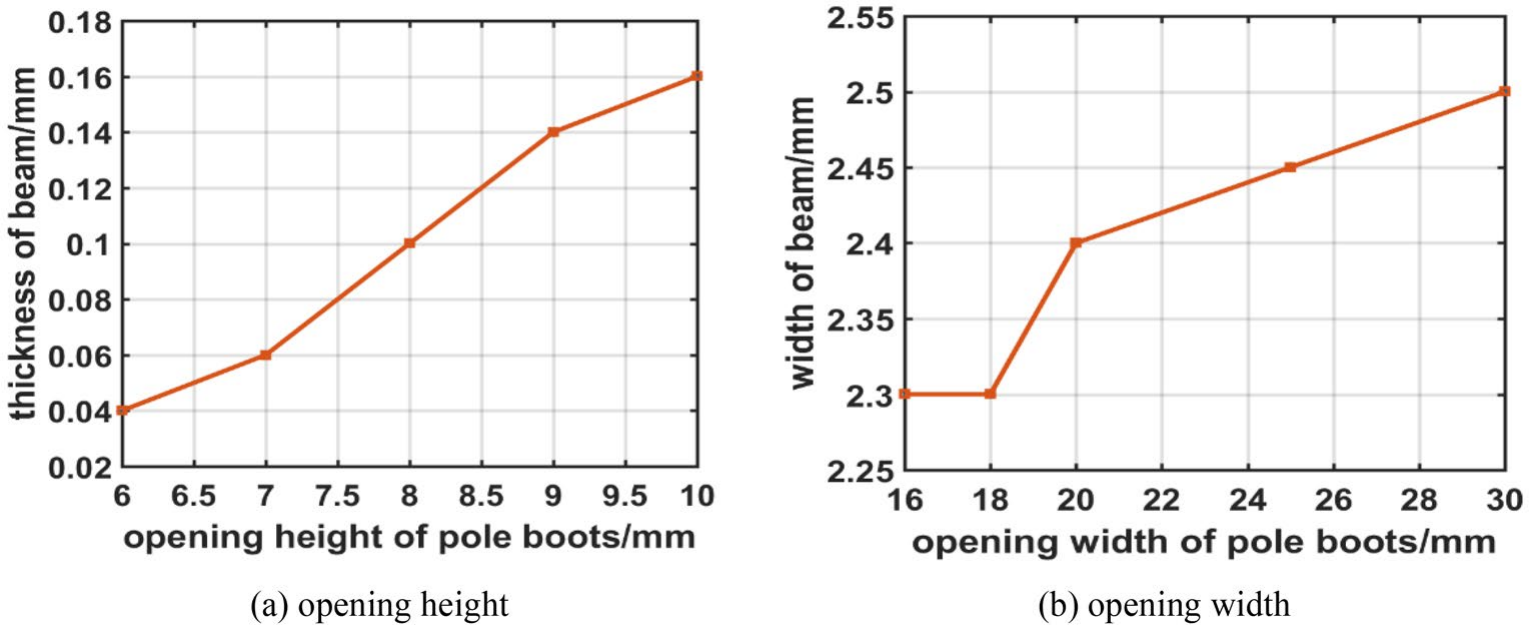
Variation of SEB size with change of pole boots (**a**) opening height and (**b**) opening width.

Figure 12 show the variations of SEB size versus pole boots height and width. In Fig. 12a, the thickness of SEB is increased as the increasing of open height of pole boots. In Fig. 12b, similarly, the width is also increased for the width of pole boots. It is because that the transverse size is enlarged, and it results to the amplitude is decreased in UPM, which produces the increases of thickness and width for the space charge effects.

### Emittance of SEB

To know the characteristics of SEB in UPM EOS, it is necessary to analyze the beam quality. Emittances are widely used to describe the beam quality in high energy accelerator. In present work, we use the emittance to describe the quality of SEB in the EOS.

For SEB, the elliptical phase space (*x*–*x*′) and (*y*–*y*′) is different. The RMS emittance in the *x*-direction of the cross-section is^13^:

3 $${\varepsilon }_{x}=\sqrt{<(x-{<x>)}^{2}>
<{\left({x}^{\mathrm{\text{'}}}-<{x}^{\mathrm{\text{'}}}>\right)}^{2}>-{<\left(x-<x>\right)\left({x}^{\mathrm{\text{'}}}-<{x}^{\mathrm{\text{'}}}>\right)>}^{2}}$$

In which, ${x}^{\mathrm{^{\prime}}}=\frac{{v}_{x}}{{v}_{normal}}$ is the orbit angle, $<x>=\frac{1}{N}\sum_{n=1}^{N}{x}_{n}$ is the normal position in x-direction.

In the *y*-direction, the emittance ${\varepsilon }_{y}$ is:

4 $${\varepsilon }_{y}=\sqrt{<(y-{<y>)}^{2}>
<{\left({y}^{\mathrm{\text{'}}}-<{y}^{\mathrm{\text{'}}}>\right)}^{2}>-{<\left(y-<y>\right)\left({y}^{\mathrm{\text{'}}}-<{y}^{\mathrm{\text{'}}}>\right)>}^{2}}$$

In which, $${\mathrm{y}}^{\mathrm{^{\prime}}}=\frac{{v}_{y}}{{v}_{normal}}$$ , $$<y>=\frac{1}{N}\sum_{n=1}^{N}{y}_{n}$$ .

**Figure 13:**
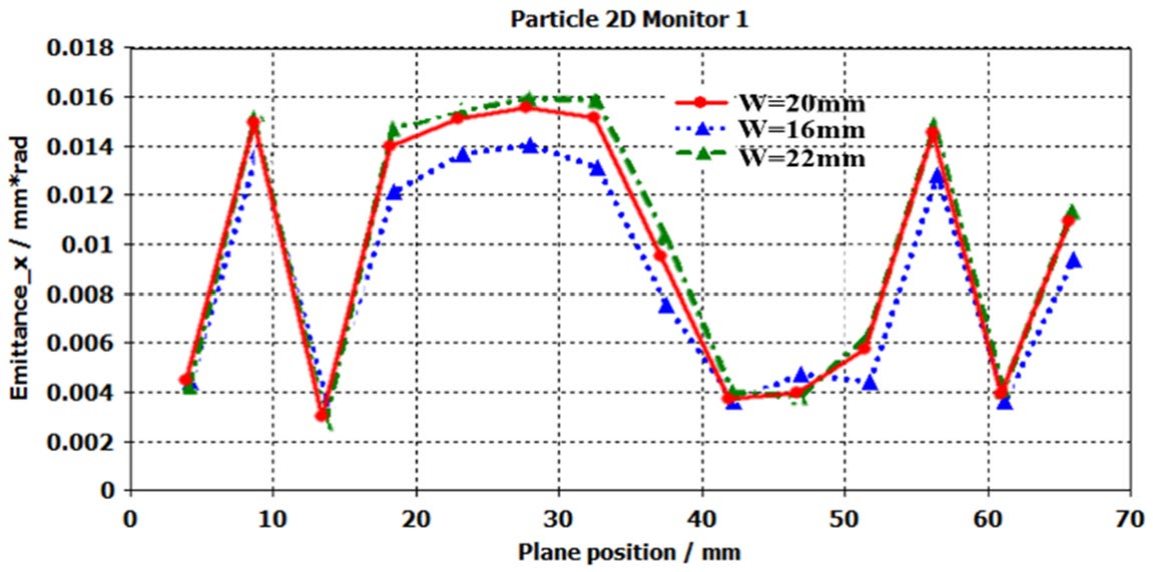
Emittance in x-direction.

Figure 13 is the emittance in *x*-direction. From the Fig. 13, we can see that the emittance is increased when the width *w* of rectangular pole boots opening, and there are some expandings in *x*-direction. The fluctuation is resulted from the space charge forces.

**Figure 14:**
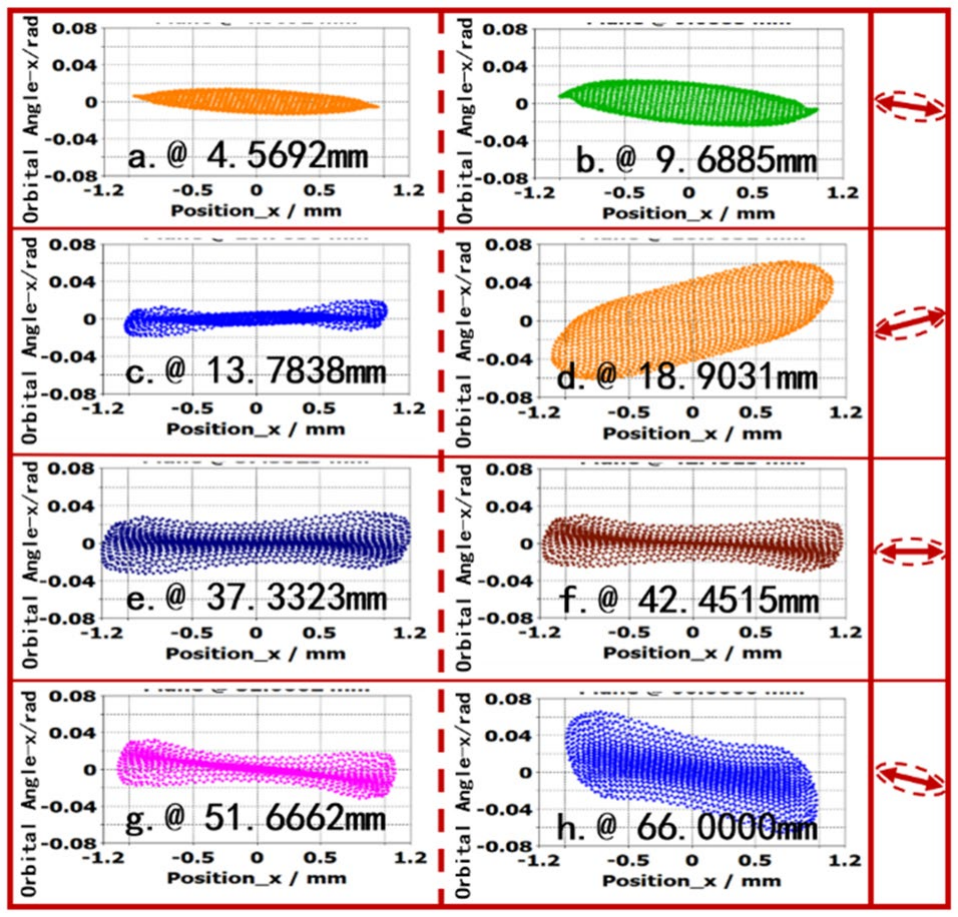
(x, *x*′) at some planes.

The varied RMS emittance ${\varepsilon }_{x}$ can be observed in elliptical phasespace as shown in Fig. 14. At the longitudinal position 4.5692 mm, the deflection of orbit angle is very small at horizontal direction, the ${\varepsilon }_{x}$ is very small which corresponds the Fig. 14. As the electron moving along with *z*-direction, the orbit angle is increased, and it reaches the maximum 0.06 rad. As the focusing magnetic field, it is adjusted the horizontal position. At the exiting of beam tunnel, the RMS is also increased as the focusing field is decreased, and the SEB is scattered in the collector.

**Figure 15:**
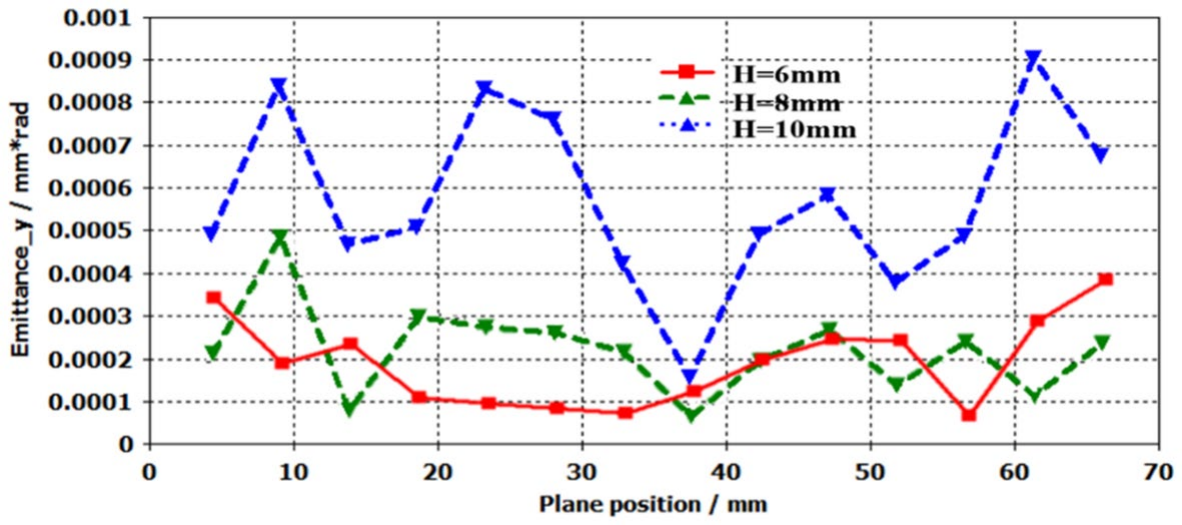
Emittance in *y*-direction.

Similarly, the transverse RMS emittance ${\varepsilon }_{y}$ in *y*-direction is also analyzed. The ${\varepsilon }_{y}$ locating different longitudinal positions is shown in Fig. 15. The results show that the changed amplitude of ${\varepsilon }_{y}$ is very small along with the longitudinal direction. However, it is varied from 3 × 10^−4^ to 9 × 10^−4^ mm rad at longitudinal position 60 mm when the height of pole boots opening is increased from 6 to 10 mm. The result shows that it is increased when the height is enlarged. To obtain the low RMS ${\varepsilon }_{y}$, we should use the thin pole boots opening height.

**Figure 16:**
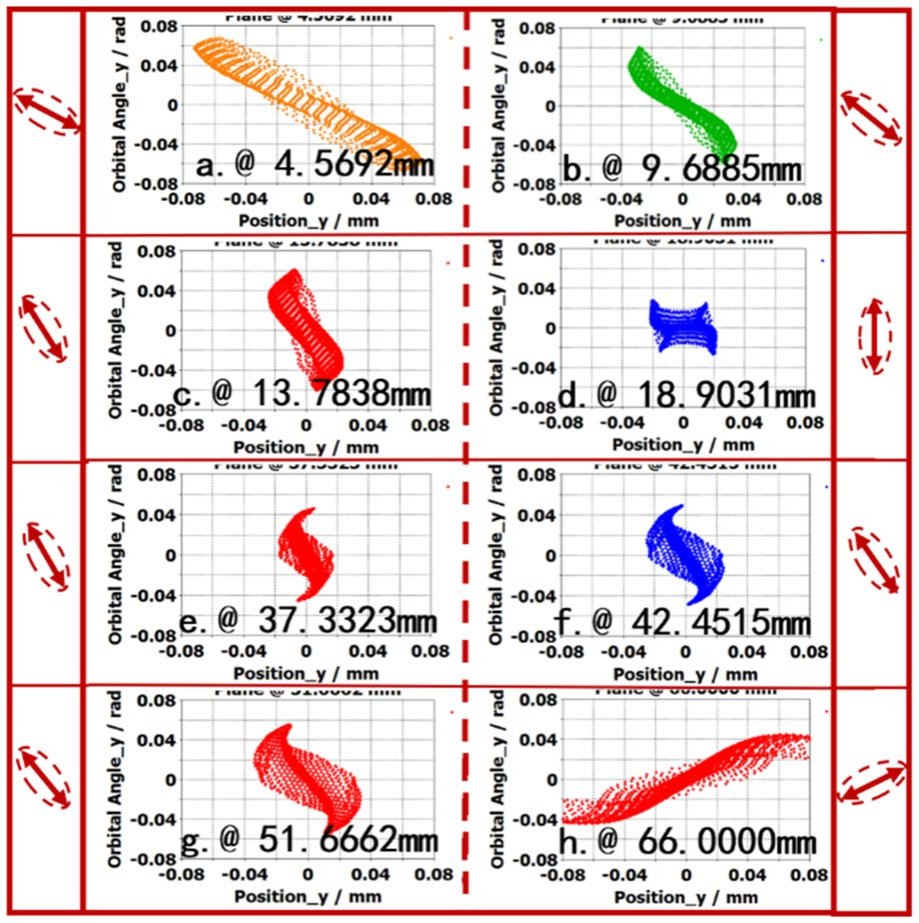
(y, *y*′) at some planes.

Figure 16 show the deflections in *y*-direction orbit angle. Seen from Fig. 16, the deflection of SEB in *y*-direction is rotated along clockwise. At the entrance of beam tunnel, the orbit angle deflection of electron beam head is 20°. At the longitudinal position of 18.9031 mm, the deflection is about 90°, which corresponds the vertical direction. At the existing of beam tunnel, the electron beam is deflected, and the deflection is about 180°. The results show that the electron beam is deflected in *y*-direction of transverse direction. Though the electron beam is deflected in *y*-direction, however, the RMS emittance ${\varepsilon }_{y}$ is very small, and the average value is about 2 × 10^−4^ mm rad, it doesn’t affected the electron beam transmission and beam-wave interaction.

**Figure 17:**
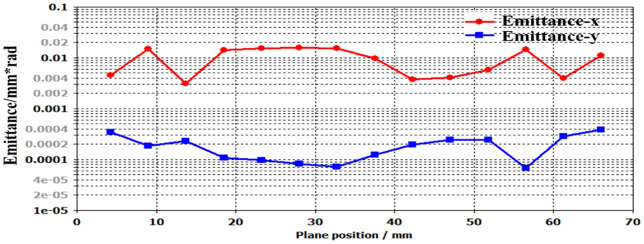
Comparisons of emittance in *x-* and *y*-direction.

To know the details of emittances in *x-*and *y-*direction, the comparisons of emittance ${\varepsilon }_{x}$ and ${\varepsilon }_{y}$ are displayed in Fig. 17, which are obtained by the width 20 mm. Seen from the results, the RMS emittance ${\varepsilon }_{x}$ is obvious larger than the ${\varepsilon }_{y}$,and the former ${\varepsilon }_{x}$ is about 100 times ${\varepsilon }_{y}$. The results are generated from the open rectangular size, the beam tunnel in *x*-direction is about 3.5 times that in *y*-direction, in which the distribution of UPM is asymmetric resulted in the remarkable changes of transverse emittance. On the other hand, the significant changes of ${\varepsilon }_{x}$ versus ${\varepsilon }_{y}$, it results in the Diocotron instability which prevents the long distances transporting of SEB in EOS. For obtaining the higher gain amplifier, it is important to keep the stability of electron beam in x-direction than that in y-direction.

## Collector for SEB

**Figure 18:**
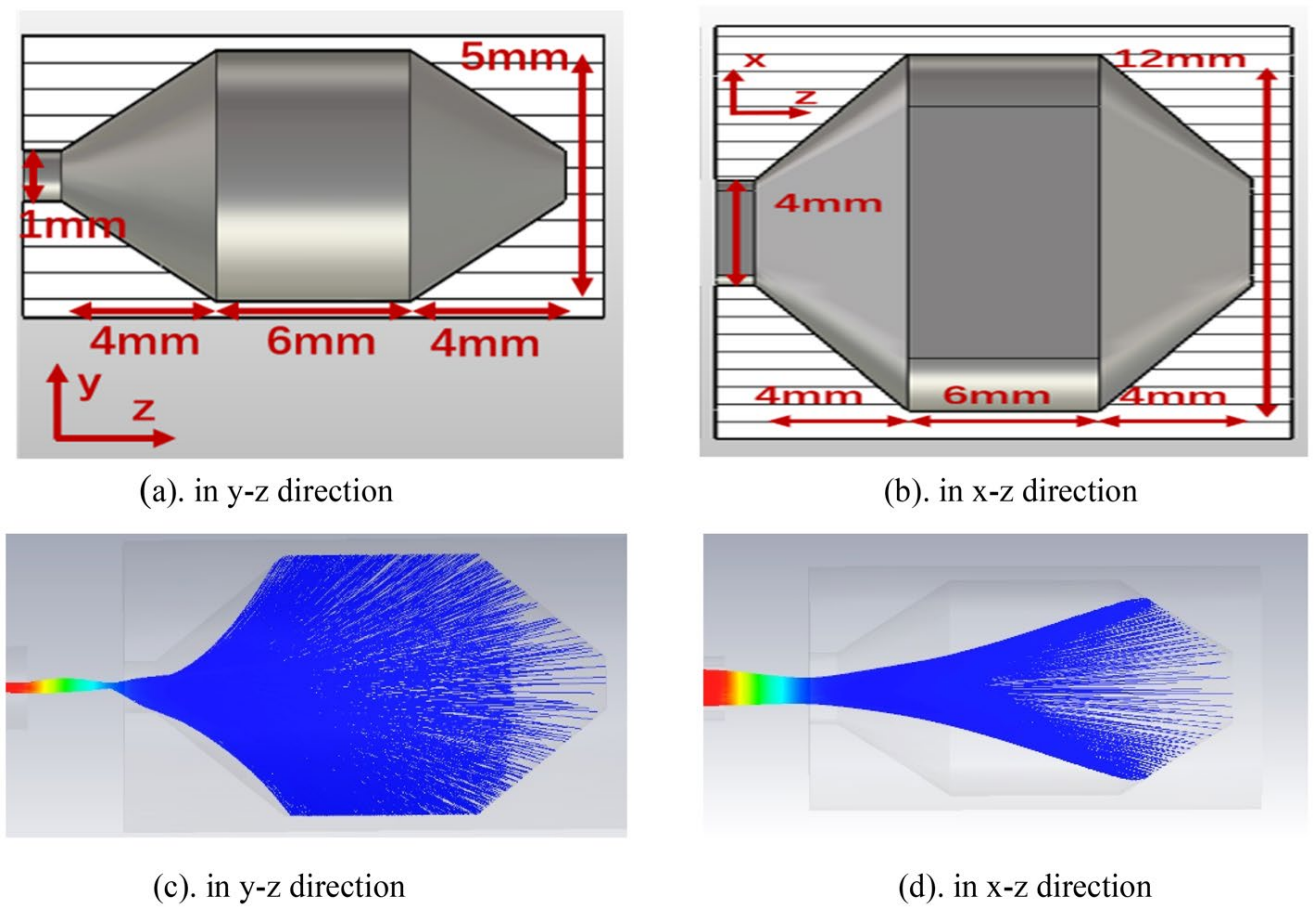
Profile of SEB Collector. (**a**) in y–z direction. (**b**) in x–z direction. The trajectories of SEB in collector (**c**) in y–z direction and (**d**) in x–z direction.

**Figure 19:**
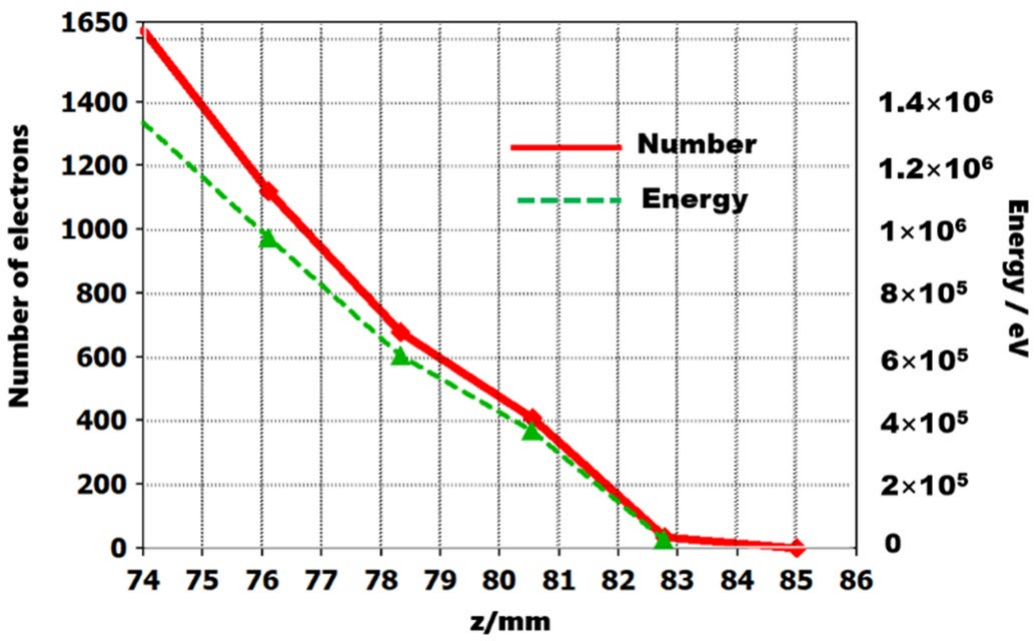
The profile of number of electrons and beam energy in z-direction in collector.

The rectangular collector for the SEB is designed as depict in Fig. 18, it is a fusiform profile which benefits to the SEB scattering and the size parameters are displayed in details. Figure 18a, b are the vertical direction in *y–z* plane and horizontal direction in *x*–*z* plane, respectively. According to the designed collector, the trajectories of SEB in collector are shown in Fig. 18, in which Fig. 18c is the vertical distribution and the Fig. 18d is the horizontal distribution. The power density of collector is 0.75 W/mm^2^, and current back streaming is 1.33%. Figure 18c,d show there is slight secondary electron emission. The variations of number of electrons and energy of beam at different longitudinal positions in collector is shown in Fig. 19, the simulated results show that the electron beam is uniformed scattering in the rectangular collector.

## Conclusion

In present paper, a SEB EOS for 0.22 THz TWT under the confining of UPM is designed. For the generation of SEB, a runway shape cathode is used, and the current emitted from the cathode is 0.3 A under the operation voltage 17 kV. Through the optimization of cathode shape, focus pole, the waist is located at 6.2 mm and thickness is about 0.035 mm. The UPM is optimized through the magnet radius, distance, height and width of pole boots opening. During the transmission of electron beam, the transverse size and emittance are analyzed, the results show that the emittance variations of ${{\varvec{\varepsilon}}}_{{\varvec{y}}}$ with 1 × 10^−4^ mm rad is less than ${{\varvec{\varepsilon}}}_{{\varvec{x}}}$ with 9 × 10^−2^ mm rad. To obtain the small transverse emittance, we should use the small width and height of pole boots opening. The deflected SEB producing by the space charge force can be adjusted under the confining of the UPM. The results show that the SEB remains a section size of 0.08 mm × 2.2 mm through the interaction region length 60 mm. It is uniformed scattering when it enters the rectangular collector. The emitted electron beam with current 0.3 A and voltage 17 kV runs 60 mm length, it is satisfied the requirements of TWT EOS operating at the frequency of 220 GHz.
